# Dataset from the Snakes (Serpentes, Reptiles) collection of the Museu Paraense Emílio Goeldi, Pará, Brazil

**DOI:** 10.3897/BDJ.7.e34013

**Published:** 2019-04-18

**Authors:** Ana Lúcia da Costa Prudente, Lorran Alves da Cruz Ramos, Timóteo Monteiro da Silva, João Fabrício de Melo Sarmento, Angelo Cortez Moreira Dourado, Fernanda Magalhães Silva, Paula Carolina Rodrigues de Almeida, Cleverson Rannieri Meira dos Santos, Marcos Paulo Alves de Sousa

**Affiliations:** 1 Museu Paraense Emílio Goeldi, Belém, Brazil Museu Paraense Emílio Goeldi Belém Brazil

**Keywords:** Snakes, collection, diversity, Amazon, inventories, biodiversity dataset, rainforest, Squamata, Serpentes, Reptiles.

## Abstract

**Background:**

We present a dataset with information from the snake collection of the Museu Paraense Emílio Goeldi, known as the “Ophidia Collection”. This collection currently has 26,728 specimens of snakes, including 9 families, 66 genera and 220 species. For the most part, it represents material from the Amazon Region. Specimens are preserved mostly in wet (alcohol) preparation, with some samples preserved in dry form, as is the case of the shells and skeletons of turtles. The dataset is now available for public consultation on the Global Biodiversity Information Facility portal (https://doi.org/10.15468/lt0wet).

**New information:**

The Herpetological collection of Museu Paraense Emílio Goeldi comprises the largest collection of its kind in the Amazon region with about 100,000 specimens of amphibians and reptiles (chelonians, alligators, lizards, snakes and amphisbaenians). This collection currently has 26,728 specimens of snakes, including 9 families, 66 genera and 220 species, some of which are endemic to the Amazon rainforest region. The Museu Paraense Emílio Goeldi is the second oldest institution of science in Brazil in activity, founded in 1866.

## Introduction

The Museu Paraense Emílio Goeldi (MPEG) or Goeldi Museum, located in Belém, Pará, Brazil, is a federal research institution within the Brazilian Ministry of Science, Technology and Communication (MCTIC). Although herpetological studies in Goeldi Museum were initiated by the Swiss naturalist Emílio Goeldi in the late 19^th^ century, the specimens collected during this period did not, however, remain in the Museum. Other important researchers contributed to the formation and enhancement of the herpetological collections, including Emília Snethlage and Gottfried Hagmann. Emília Snethlage was hired at the Goeldi Museum in June 1905, when she began developing numerous field works on scientific expeditions in the Amazon to collect specimens. In 1914, she became director of the Goeldi Museum, being the first woman to administer a scientific institution in South America (Junghans 2008).

In June 1965, a Division of Herpetology was installed at the Goeldi by Osvaldo R. Cunha. The rapid increase in knowledge of the herpetofauna from the 1950s to 1975 was largely associated with the work of Osvaldo R. Cunha and Francisco P. Nascimento, who developed an ambitious field collection programme (Cunha and Nascimento 1978;Cunha and Nascimento 1993; Hoogmoed et al. 2011). This reptile collection initiative included collaboration with inhabitants of several localities, initially covering the eastern region of Pará, later extending to the south of the state and to the west of Maranhão, Brazil. This long-term study resulted in a series of important papers (as listed in Hoogmoed et al. 2011), which became basic reference works for the eastern Amazonian snakes.

Despite this important sampling effort by Cunha and Nascimento, accessibility and time constraints hindered full coverage of the area, such that many municipalities remained unsampled. More recently, detailed field studies of some of these areas, plus the inclusion of new areas, has increased our knowledge of the herpetological fauna of the region (Avila-Pires 1995; Avila-Pires et al. 2009; Rodrigues and Prudente 2011; Silva et al. 2011; Prudente et al. 2013; Prudente et al. 2018). The Herpetological collection of the MPEG constitutes an irreplaceable source of information for these studies and represents an historical record for the occurrence of several species in areas that are now totally altered by anthropic actions, especially in the region known as the “arc of deforestation” (Prudente et al. 2018).

**Figure 1.**
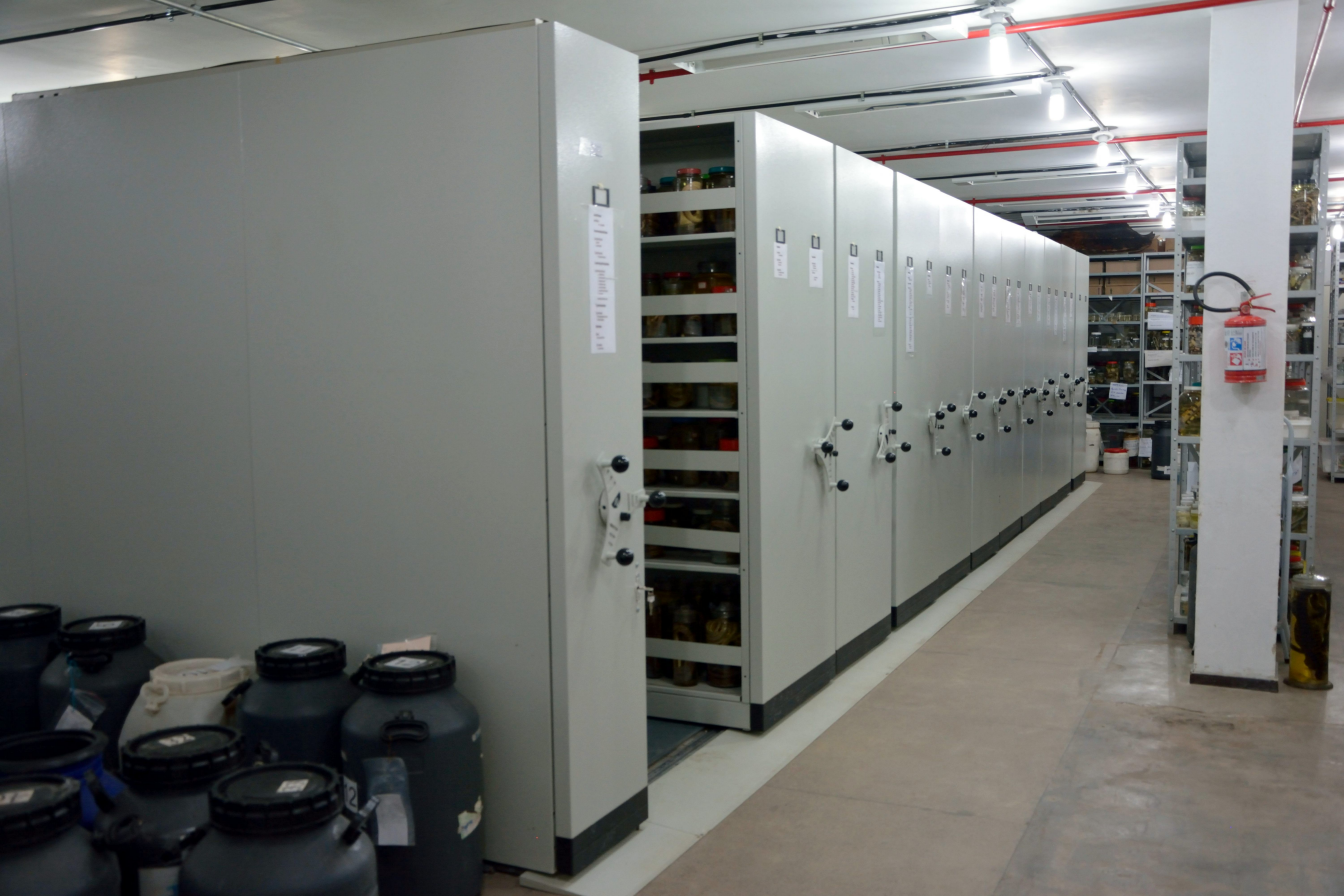
General view of the Herpetological collection of the Museu Paraense Emílio Goeldi, Belém, Pará, Brazil.

Currently, the Herpetological collection comprises the largest collection of its kind in the Amazon region, with about 100,000 specimens of amphibians and reptiles (chelonians, alligators, lizards, snakes and amphisbaenians) (Fig. 1). There are currently three staff researchers and one associate researcher, as well as undergraduate and graduate students from the Postgraduate Program in Zoology (PPGZOOL) of the Federal University of Pará/MPEG and in the recently created graduate programme in Biodiversity and Evolution (PPGBE) at MPEG. In this paper, we describe and synthesise information about Amazonian snake biodiversity as represented in the collection of MPEG, by providing a summary of taxonomic coverage and geographical distribution, in the hopes of facilitating rapid and dynamic access to these records.

Data published through GBIF: https://doi.org/10.15468/lt0wet.

## Sampling methods

### Sampling description

The specimens were preserved mostly in liquid (alcohol) collections, although some individuals were preserved as dry specimens. The collection also had tissue samples for molecular studies.

### Quality control

The snake collection of MPEG has received collections from dozens of scientists who had used various methods of sampling.They included time-constrained search, pitfall traps with drift fence and incidental encounters (Santos-Costa and Prudente 2003; Maschio et al. 2016). The validity of species’ names was checked using the catalogue The Brazilian Reptiles – List of species (Costa and Bérnils 2018). Synonymies were checked across all mentioned lists and, if incongruences were found, the earliest name on the record was used for disambiguation. If the names used in the earlier lists did not resolve the nomenclatural inconsistency, geographical ranges of the species were checked and used to assign the currently accepted species name. If identification problems persisted after these steps, the authors carried out a more thorough re-identification of the specimens. Given the current data policy of the Goeldi Museum biological collections, collection sites without specific geographic coordinates, identified with locality names, were georeferenced using the location of the municipal centre of the region of occurrence, in order to guarantee a consistent distribution for Occurrence Data of Sensitive Primary Species.

## Geographic coverage

### Description

**Figure 2.**
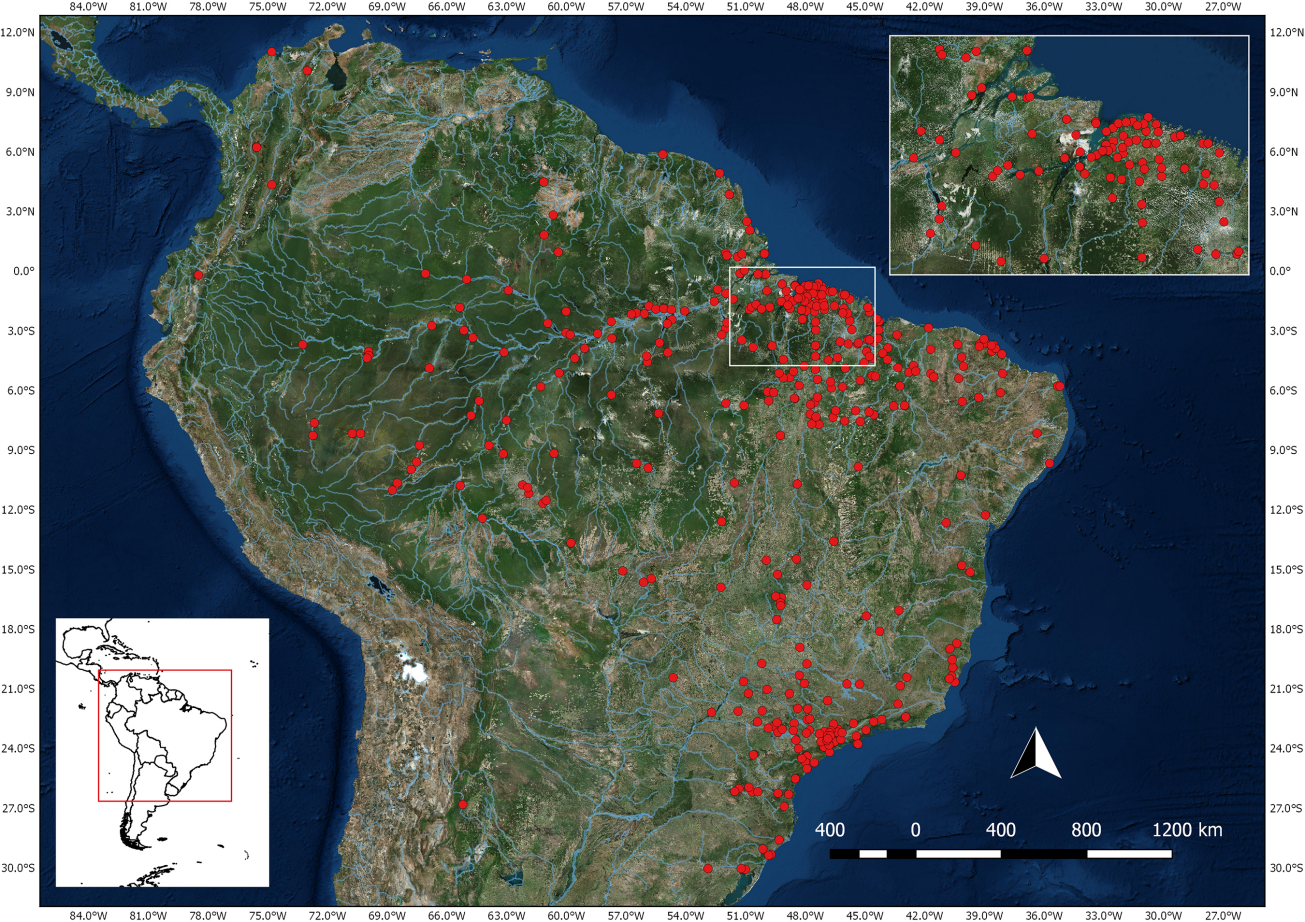
Map of collection occurrence records, represented by sampled municipality, where one point may represent more than one event. Most specimens were captured within the rectangle area.

Although most of the species came from Brazil (n= 26,678), there are records for Argentina (n= 1), Colombia (n= 27), Ecuador (n= 1), French Guiana (n= 1), Peru (n= 1) and Suriname (n= 1) (Fig. 2. Most samples from Brazil were from the Amazon region, mainly in eastern Pará state. Most specimens were captured within the polygon defined by the following coordinates: 1°0'0"N and 4°0'0"S latitude; 51°0'0"W and 45°0'0"W longitude. Coordinates 12°0'0"N and 30°0'0"S latitude; 81°0'0"W and 33°0'0"W longitude.

## Taxonomic coverage

### Description

The snakes collection of MPEG includes 26,728 specimens, representing 9 families, 66 genera and 220 species. About 99% of the specimens come from Brazil, with few records in Colombia (27 specimens), Argentina (2 specimens), Ecuador, French Guiana, Peru and Suriname (with one specimen each country). For the most part, this material came from the Amazon. There are 15 holotypes, one neotype (*Hydrodynastes bicintus*) and 13 paratypes. More than 98% of the specimens are identified at the species level.

**Figure 3.**
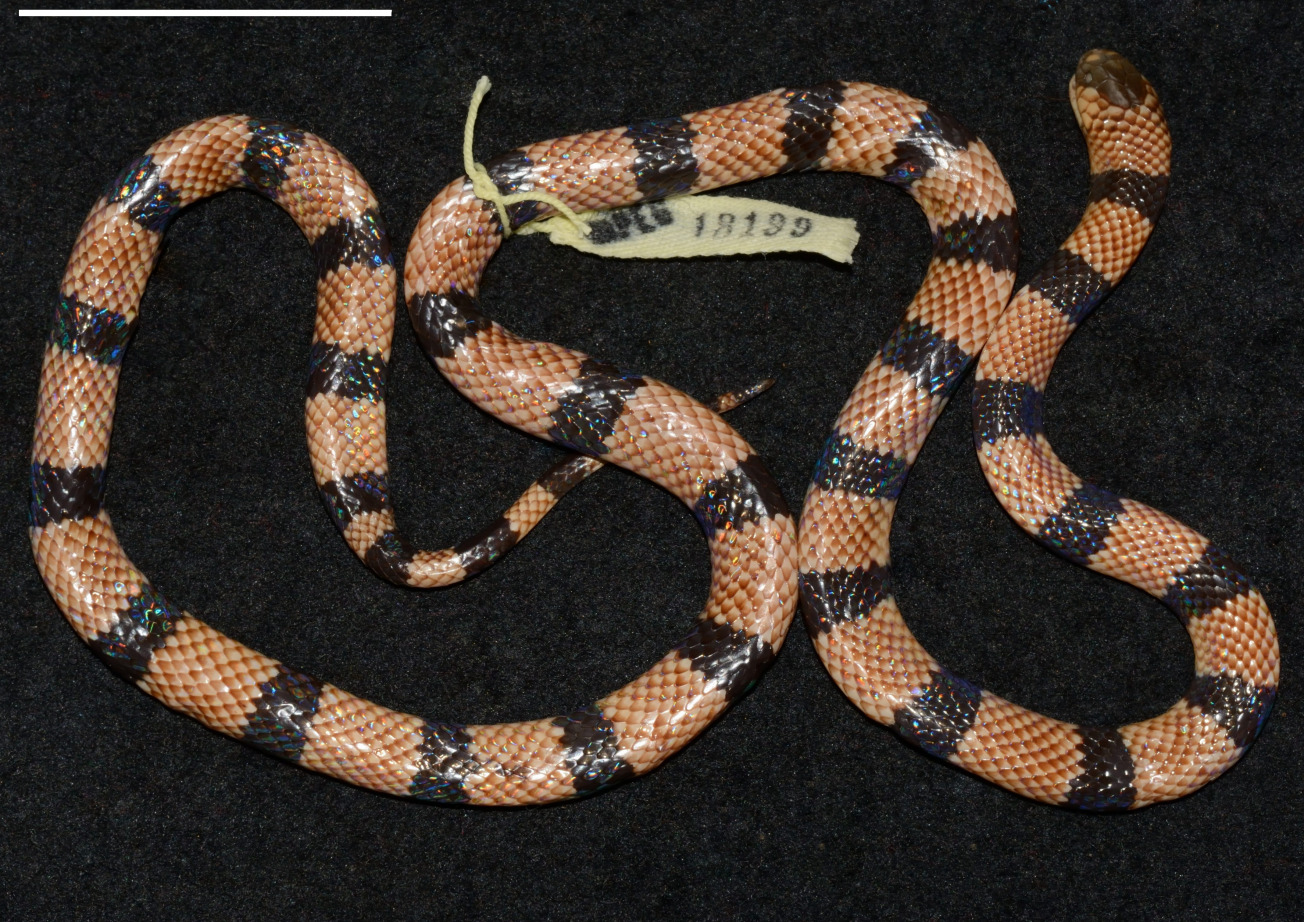
Dorsal view of the holotype of *Micrurus tikuna* (MPEG 18199), from the municipality of Tabatinga (04º14'36'S, 69º54'15'W), state of Amazonas, Brazil. Scale = 50 mm

All type species found in the collection are detailed below:

List of species with holotype in the collection:

*Atractus albuquerquei* Cunha & Nascimento, 1983; *Atractus alphonsehogei* Cunha & Nascimento, 1983; *Atractus boimirim* Passos, Prudente & Lynch, 2016; *Atractus caxiuana* Prudente & Santos-Costa, 2006; *Atractus snethlageae* Cunha & Nascimento, 1983; *Atractus hoogmoedi* Prudente & Passos, 2010; *Atractus natans* Hoogmoed & Prudente, 2003; *Atractus surucucu* Prudente & Passos, 2008; *Erythrolamprus carajasensis* Cunha, Nascimento & Avila-Pires, 1985; *Mastigodryas bifossatus lacerdai* Cunha & Nascimento, 1978; *Micrurus paraensis* Cunha & Nascimento, 1973; *Micrurus tikuna* Feitosa, Silva Jr, Pires, Zaher & Prudente, 2015 (Fig. 3); *Oxyrhopus melanogenys orientalis* Cunha & Nascimento, 1983; *Sibynomorphus mikanii septentrionalis* Cunha et al 1980; *Taeniophallus quadriocellatus* Santos-Jr, Di-Bernardo & Lema, 2008.

List of species with only paratypes in the collection:

*Atractus alphonsehogei* (n = 6), *Atractus boimirim* (n = 15), *Atractus caxiuana* (n = 3), *Atractus hoogmoedi* (n = 2), *Atractus natans* (n = 2), *Atractus snethlageae* (n = 13), *Atractus surucucu* (n = 2), *Atactus tartarus* Passos, Prudente & Lynch, 2016 (n = 6); *Erythrolamprus carajasensis* (n = 27), *Micrurus paraensis* (n = 2), *Oxyrhopus melanogenys orientalis* (n = 94), *Sibynomorphus mikanii septentrionalis* (n = 41), *Taeniophallus quadriocellatus* (n = 5).

**Taxonomic ranks**

**Kingdom**: Animalia

**Phylum**: Chordata

**Class**: Reptilia

**Order**: Squamata

**Figure 4.**
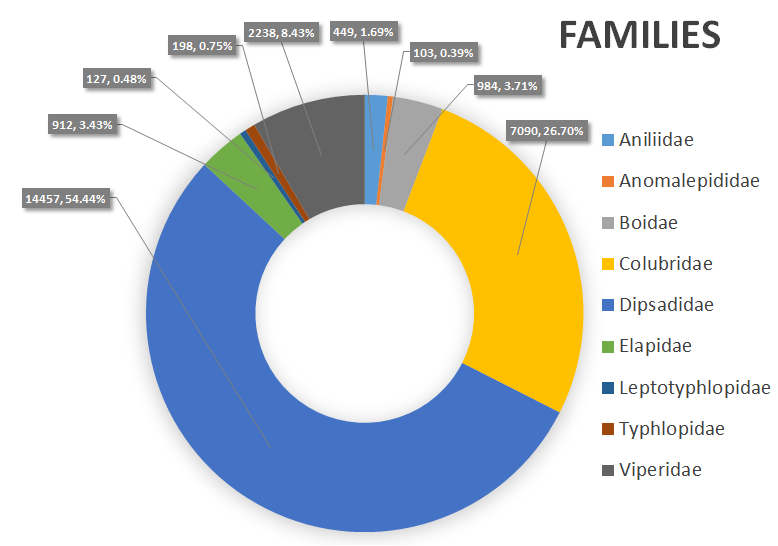
Distribution of families in the snake collections, MPEG. Number of specimens and frequencies are represented for each family.

**Family**: Aniliidae, Anomalepididae, Boidae, Colubridae, Dipsadidae, Elapidae, Leptotyphlopidae, Typhlopidae and Viperidae (Fig. 4).

## Temporal coverage

**Formation period:** 1900-2017.

### Notes

**Figure 5.**
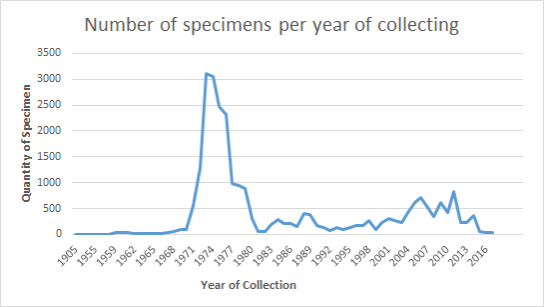
Distribution of the number of specimens collected per year in the snake collection, MPEG

The temporal range of the records is between 1900–2017 (Fig. 5). A rapid increase in knowledge of the eastern Amazonian herpetofauna from the 1950s to 1975 was largely associated with the work of Osvaldo R. Cunha and Francisco P. Nascimento, MPEG researchers who developed a collection programme in the region during that period (Hoogmoed et al. 2011). Despite significant sampling efforts by several collaborators, accessibility and time constraints hindered full coverage of the area, such that many municipalities remained unsampled.

## Collection data

### Collection name

Ophidia

### Collection identifier

MPEG.HOP

### Parent collection identifier

Museu Paraense Emílio Goeldi

### Specimen preservation method

Alcohol

## Usage rights

### Use license

Other

### IP rights notes

Creative Commons Attribution Non Commercial (CC-BY-NC) 4.0 License

## Data resources

### Data package title

Museu Paraense Emílio Goeldi - Ophidia Collection

### Resource link

https://www.gbif.org/dataset/85bce05a-fce5-4918-a8f0-ab4b82718c25

### Alternative identifiers

https://doi.org/10.15468/lt0wet

### Number of data sets

1

### Data set 1.

#### Data set name

Museu Paraense Emílio Goeldi - Ophidia Collection

#### Data format

Darwin Core Archive (DwC-A)

#### Number of columns

28

#### Download URL

https://www.gbif.org/dataset/85bce05a-fce5-4918-a8f0-ab4b82718c25

#### Data format version

11.2

#### Description

This collection currently has 26,728 specimens of snakes, including 9 families, 66 genera and 220 species. The full database is available via the Integrated Publishing Toolkit (IPT) of Museu Paraense Emílio Goeldi (version 3.18 published in 2019-03-26).

**Data set 1.** 

Column label	Column description
occurrenceID	An identifier for the Occurrence
dcterms:modified	The most recent date-time on which the resource was changed
dcterms:license	A legal document giving official permission to do something with the resource
dcterms:rightsHolder	A person or organisation owning or managing rights over the resource
institutionCode	The name (or acronym) in use by the institution having custody of the object(s) or information referred to in the record
collectionCode	The name, acronym, coden or initialism identifying the collection or dataset from which the record was derived
datasetName	The name identifying the dataset from which the record was derived
basisOfRecord	The specific nature of the data record - a subtype of the dcterms:type
catalogNumber	An identifier for the record within the dataset or collection
recordedBy	A list of names of people, groups or organisations responsible for recording the original Occurrence
preparations	A list of preparations and preservation methods for a specimen
otherCatalogNumbers	A list of previous or alternate fully qualified catalogue numbers or other human-used identifiers for the same Occurrence
EventDate	The date-time or interval during which an Event occurred
higherGeography	A list of geographic names less specific than the information captured in the locality term.
continent	The name of the continent in which the Location occurs
country	The name of the country or major administrative unit in which the Location occurs
stateProvince	The name of the next smaller administrative region than country
county	The full, unabbreviated name of the next smaller administrative region than stateProvince
typeStatus	A list of nomenclatural types
scientificName	The full scientific name
kingdom	The full scientific name of the kingdom in which the taxon is classified
phylum	The full scientific name of the phylum or division in which the taxon is classified
class	The full scientific name of the class in which the taxon is classified
order	The full scientific name of the order in which the taxon is classified
family	The full scientific name of the family in which the taxon is classified
genus	The full scientific name of the genus in which the taxon is classified
specificEpithet	The name of the first or species epithet of the scientificName
infraspecificEpithet	The name of the lowest or terminal infraspecific epithet of the scientificName, excluding any rank designation

## Additional information

### Data publication protocol

**Figure 6.**
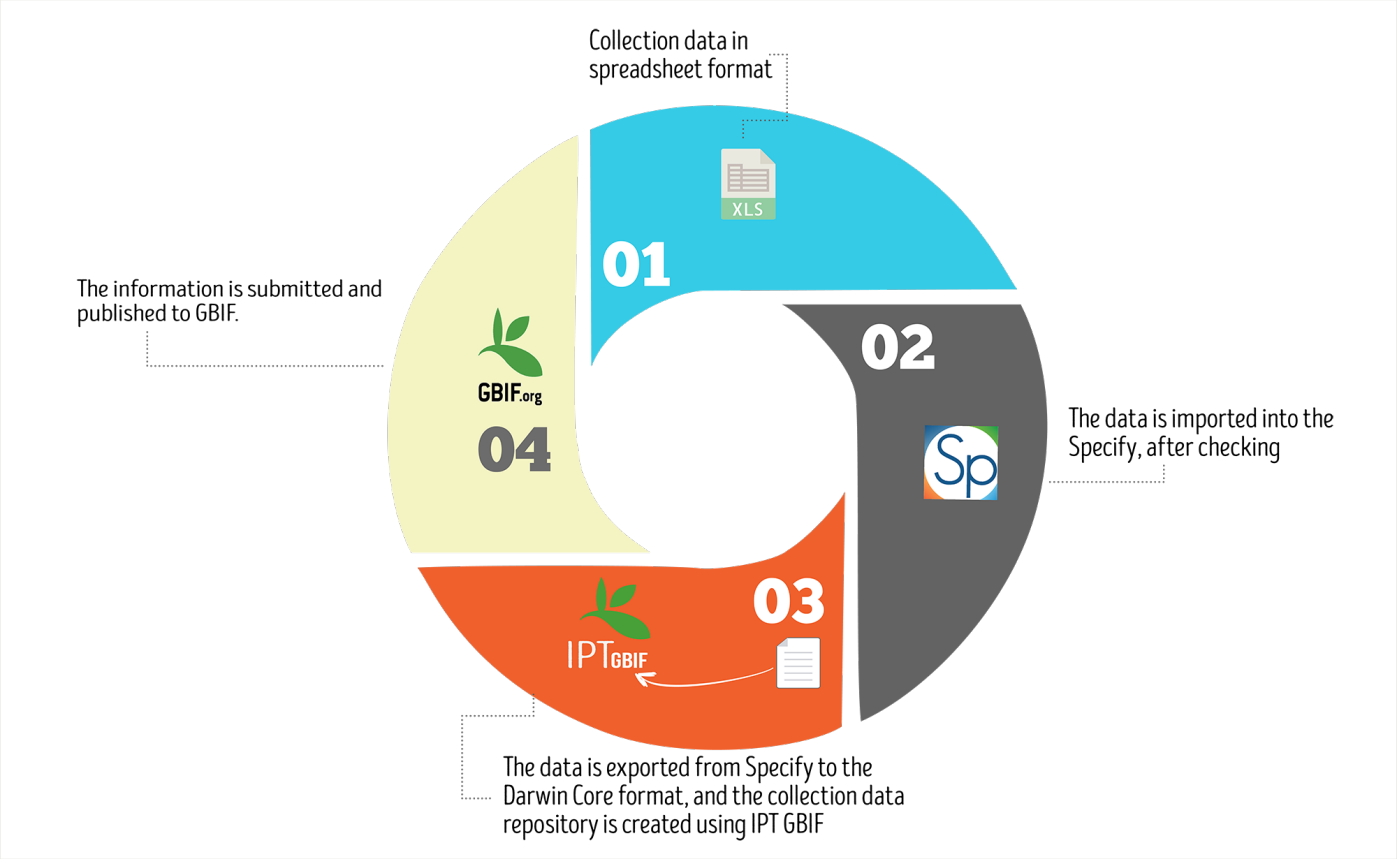
Schematic diagram of data publication protocol.

Prior to digitising the collection, the preservation status of specimens was reviewed. Specimens were identified or existing identifications were reviewed. The digitising and publication process followed the protocols of previous work in the Goeldi's Ichthyology collection (Silva et al. 2017), as illustrated in Fig. 6. First, all biodiversity and biological collection data were digitised in a Microsoft Excel spreadsheet adopting the Specify format (SPECIFY 2018). Next, the data spreadsheet was imported into the Specify database, using the Workbench tool to perform a data check for duplicate records, consistency and standardisation errors (for example, geographical coordinates, date etc.). After this data check, the data were imported. Then the data were exported from Specify software to the Darwin Core Archive format v1.4 (Wieczorek et al. 2012), creating a dataset with metadata. In the fourth and final step, a collection dataset repository was created using the Integrated Publishing Toolkit (IPT), which was submitted and published in GBIF (http://www.gbif.org).

### Curatorship and storage

The material is identified by comparison with bibliographic sources and material present in the collection. The data and metadata are digitised and deposited in the collection and maintained in air-conditioning at 22°C. The specimens are fixed in formalin for 24 hours and transferred into a 70% ethanol solution for permanent storage. Snakes are injected at 4-5 cm intervals along the whole length of belly and tail. Moderate pressure at the base of the tail of a freshly killed snake everts its hemipenes. Hemipenial morphology is very helpful in taxonomic determinations. Injection of formalin at the tail base also serves to put pressure to evert hemipenis and harden them at the same time. Samples are stored in glass jars or other containers (for example, high density polyethylene drums), organised in alphabetical order by family, genus and species. The tissue samples are taken from freshly killed specimens, preserved in ethanol and stored in a freezer. Loans, exchanges and donation of materials are made through a request to the curator, who evaluates each proposal.
